# Spectrum of germline pathogenic variants using a targeted next generation sequencing panel and genotype-phenotype correlations in patients with suspected hereditary breast cancer at an academic medical centre in Pakistan

**DOI:** 10.1186/s13053-022-00232-2

**Published:** 2022-06-16

**Authors:** Fizza Akbar, Zahraa Siddiqui, Muhammad Talha Waheed, Lubaina Ehsan, Syed Ibaad Ali, Hajra Wiquar, Azmina Tajuddin Valimohammed, Shaista Khan, Lubna Vohra, Sana Zeeshan, Yasmin Rashid, Munira Moosajee, Adnan Abdul Jabbar, Muhammad Nauman Zahir, Naila Zahid, Rufina Soomro, Najeeb Niamat Ullah, Imran Ahmad, Ghulam Haider, Uzair Ansari, Arjumand Rizvi, Arif Mehboobali, Abida Sattar, Salman Kirmani

**Affiliations:** 1 Division of Women and Child Health, Aga Khan University, Karachi, Pakistan; 2 Medical College, Aga Khan University, Karachi, Pakistan; 3 School of Medicine, Western Michigan University Stryker, Kalamazoo, MI USA; 4 Department of Oncology, Aga Khan University, Karachi, Pakistan; 5 Department of Surgery, Aga Khan University, Karachi, Pakistan; 6 Liaquat National Hospital and Medical College, Karachi, Pakistan; 7 Shaukat Khanum Hospital, Karachi, Pakistan; 8 Cancer Foundation Hospital, Karachi, Pakistan; 9 Bait-Ul-Sukoon Cancer Hospital, Karachi, Pakistan; 10 Department of Pediatrics and Child Health, Aga Khan University, Karachi, Pakistan

**Keywords:** Hereditary breast Cancer, Germline pathogenic variant, Consensus guidelines, Genetic counseling, Genetic testing, Genetic services manuscript

## Abstract

**Background:**

Breast cancer is the most common malignancy in women, affecting over 1.5 million women every year, which accounts for the highest number of cancer-related deaths in women globally.

Hereditary breast cancer (HBC), an important subset of breast cancer, accounts for 5–10% of total cases. However, in Low Middle-Income Countries (LMICs), the population-specific risk of HBC in different ethnicities and the correlation with certain clinical characteristics remain unexplored.

**Methods:**

Retrospective chart review of patients who visited the HBC clinic and proceeded with multi-gene panel testing from May 2017 to April 2020.

Descriptive and inferential statistics were used to analyze clinical characteristics of patients. Fisher’s exact, Pearson’s chi-squared tests and Logistic regression analysis were used for categorical variables and Wilcoxon rank-sum test were used for quantitative variables. For comparison between two independent groups, Mann-Whitney test was performed. Results were considered significant at a *p* value of < 0.05.

**Results:**

Out of 273 patients, 22% tested positive, 37% had a VUS and 41% had a negative genetic test result. Fifty-five percent of the positive patients had pathogenic variants in either *BRCA1* or *BRCA2*, while the remaining positive results were attributed to other genes. Patients with a positive result had a younger age at diagnosis compared to those having a VUS and a negative result; median age 37.5 years, IQR (Interquartile range) (31.5–48). Additionally, patients with triple negative breast cancer (TNBC) were almost 3 times more likely to have a positive result (OR = 2.79, CI = 1.42–5.48 *p* = 0.003). Of all patients with positive results, 25% of patients had a negative family history of breast and/or related cancers.

**Conclusions:**

In our HBC clinic, we observed that our rate of positive results is comparable, yet at the higher end of the range which is reported in other populations. The importance of expanded, multi-gene panel testing is highlighted by the fact that almost half of the patients had pathogenic or likely pathogenic variants in genes other than *BRCA1/2*, and that our test positivity rate would have only been 12.8% if only *BRCA1/2* testing was done. As the database expands and protocol-driven referrals are made across the country, our insight about the genetic architecture of HBC in our population will continue to increase.

**Supplementary Information:**

The online version contains supplementary material available at 10.1186/s13053-022-00232-2.

## Introduction

Breast cancer is the most common malignancy worldwide, affecting over 1.5 million women (25% of all women with cancer) every year, accounting for the highest number of cancer-related deaths in women globally [1, 2].

The risk of developing breast cancer is broadly categorized into non-hereditary and hereditary cancer risk. The baseline population risk of having a diagnosis of sporadic breast cancer in a woman ranges between 10 and 12%, which means that roughly one in nine women will develop breast cancer in their lifetime [3]. This risk substantially increases in individuals with a germline disease-causing variant in one of the breast cancers associated genes, confirming a diagnosis of hereditary breast cancer (HBC).

HBC, an important subset of breast cancer, accounts for 5–10% of total cases [1, 4, 5]. These cases are attributed to pathogenic (P) and likely pathogenic (LP) germline variants in genes that cause a predisposition for breast cancer [6, 7]. In addition, approximately 20% of individuals with breast cancer have a close relative who also had breast cancer, suggesting a familial link, but no specific genetic variant is identified [8].

Identifying HBC is crucial for optimizing long-term outcomes in both symptomatic and pre-symptomatic individuals. Patients diagnosed with a hereditary breast cancer syndrome are eligible for tailored management, based on whether they have a high or moderate risk of developing breast or other related cancers in the future. They can, thus be offered a more precise and individualized management plan, that can include optimal surgical intervention and eligibility for targeted therapy, along with the need for a high-risk surveillance plan or/and prophylactic surgery for associated malignancy risk, summarized in Table 1 [9].

**Table 1:** NCCN recommendation for gene specific risk assessment, high risk surveillance plan and prophylactic surgery for associated malignancy risk. Table adapted from NCCN Guidelines (version 2.2021) [9]

Gene	Evidence for increased risk	Absolute risk (%)	Other cancer risks and syndromes	Breast cancer management	Other cancers management
*BRCA1*	Very strong (with predisposition to triple-negative disease)	> 60	Ovarian cancer Pancreatic cancer Prostate cancer	Breast awareness starting at 18 months Clinical breast exam, every 6–12 months, starting at 25 years of age Annual breast MRI screening Discuss option of RRM Annual mammogram screening in men with gynecomastia at age 50 or 10 years before the earliest known male breast cancer in the family	Recommend RRBSO typically at 35–40 years of age If no RRBSO, then TVUS with serum CA-125 for ovarian cancer screening starting at 30–35 years Consider prostate cancer screening in men starting at age 40 years Pancreatic cancer screening using contrast enhanced MRI/MRCP or EUS
*BRCA2*	Very strong (with predisposition to ER+ disease)	> 60	Ovarian cancer Pancreatic cancer Prostate cancer Melanoma	Breast awareness starting at 18 months Clinical breast exam, every 6–12 months, starting at 25 years of age Annual breast MRI screening Discuss option of RRM Annual mammogram screening in men with gynecomastia at age 50 or 10 years before the earliest known male breast cancer in the family	Recommend RRBSO typically at 40–45 years of age If no RRBSO, then TVUS with serum CA-125 for ovarian cancer screening starting at 30–35 years Recommend prostate cancer screening in men starting at age 40 years Pancreatic cancer screening using contrast enhanced MRI/MRCP or EUS
*ATM*	Strong	15–40	Ovarian cancer Pancreatic cancer Ataxia telangiectasia	Screening with annual mammogram with consideration for tomosynthesis and consider breast MRI with contrast starting at age 40 years Insufficient evidence for RRM, manage based on FH	Insufficient evidence for RRBSO, manage based on FH Screening mutation carriers with a family history of pancreatic cancer
*CHECK2*	Strong	15–40	Colon cancer	Screening with annual mammogram with consideration for tomosynthesis and consider breast MRI with contrast starting at age 40 years Insufficient evidence for RRM, manage based on FH	Colon cancer surveillance is recommended same as APC which is colonoscopy (preferred) or flexible sigmoidoscopy every 12 months starting at age 10–15 years
*NF1*	Strong	15–40	Malignant peripheral nerve sheath tumors, GIST, others	Screening with annual mammogram with consideration for tomosynthesis starting at age 30 years and consider breast MRI with contrast from ages 30–50 years Insufficient evidence for RRM, manage based on FH	Recommend referral to NF1 specialist for evaluation and management of other associated tumors
*CDH1*	Strong	41–60	Hereditary diffuse gastric cancer	Screening with annual mammogram with consideration for tomosynthesis and consider breast MRI with contrast starting at age 30 years Insufficient evidence for RRM, manage based on FH	Prophylactic total gastrectomy for CDH1 mutation carriers is recommended between ages 18 and 40 years and earlier than 18 years if gastric cancer in a family member before 25 years of age If patient elects not to undergo gastrectomy, screen every 6–12 months by upper endoscopy with multiple random biopsies
*PALB2*	Strong	41–60	Ovarian cancer Pancreatic cancer (limited increased risk)	Screening with annual mammogram with consideration for tomosynthesis and breast MRI with contrast starting at age 30 years Discuss option of RRM	For ovarian cancer management there is insufficient evidence, managed based on FH Screening mutation carriers with a family history of pancreatic cancer
*PTEN*	Strong	41–60	Thyroid cancer Colon cancer Endometrial cancer Cowden syndrome	Women: Screening with annual mammogram with consideration for tomosynthesis and breast MRI with contrast starting at age 30–35 years or 5–10 years before the earliest known breast cancer in the family Discuss option for RRM	Start endometrial cancer screening by age 35 years, patient education regarding AUB and symptoms is important, consider endometrial biopsy every 1–2 years Discuss option of hysterectomy upon completion of family Men and Women: Annual thyroid U/S starting at age 7 years Colonoscopy starting at age 35 years unless symptomatic or if close relative with colon cancer, then start 5–10 years before the earliest known colon cancer in the family Consider renal U/S starting at age 40 years, then every 1–2 year Annual dermatology exams for melanoma
*STK11*	Strong	41–60	Ovarian (non-epithelial) cancer Pancreatic cancer Peutz-Jeghers syndrome	Evidence insufficient for RRM, manage based on FH Peutz-Jeghers syndrome management: Clinical breast exam every 6 months, with annual mammogram and breast MRI starting at 30 years of age	Colonoscopy and upper endoscopy every 2–3 years starting in late teens Small bowel visualization (CT or MRI enterography or video capsule endoscopy baseline starting at age 8–10 years, then every 2–3 years Pancreatic cancer screening using contrast enhanced MRI/MRCP or EUS Annual physical examination for observation of precocious puberty starting at 8 years Pelvic examination and pap smear annually starting 18–20 years Annual testicular exam and observation for feminizing changes starting at 10 years
*TP53*	Strong	> 60	Pancreatic cancer (limited) Li-Fraumeni syndrome	Screening with annual mammogram with consideration for tomosynthesis and breast MRI with contrast Discuss option of RRM	Colonoscopy and upper endoscopy every 2–5 years starting at 25 years or 5 years before the earliest known colon cancer in the family Annual dermatological examination starting at 18 years Annual whole-body MRI including brain Screening mutation carriers with a family history of pancreatic cancer
*BARD1*	Limited (but stronger with triple negative disease)	Insufficient data	None	Screening with annual mammogram with consideration for tomosynthesis and consider breast MRI with contrast starting at age 40 Insufficient evidence for RRM, manage based on FH	
*BRIP1*	Limited; potential increase in female breast cancer (including triple negative disease)	Insufficient data	Ovarian cancer	Insufficient data for breast cancer management, managed based on FH	Consider RRSO at 45–50 years of age
*NBN*	No increased risk except 657del5 mutation which has mixed evidence	Insufficient data	Limited risk for Ovarian cancer	Insufficient data for breast cancer management, managed based on FH	Ovarian cancer management also based on FH
*MSH2* *MLH1* *MSH6* *PMS2* *EPCAM*	Limited	< 15	Ovarian cancer Pancreatic cancer (excluding PMS2) Colon, Uterine and others Lynch syndrome	Insufficient data for breast cancer management, managed based on FH	Screening mutation carriers with a family history of pancreatic cancer Other management is under Lynch syndrome management requires surveillance in accordance with the mutated gene
*RAD51C*	Limited; potential increase in female breast cancer (including triple negative disease)	15–40	Ovarian cancer	Insufficient data for breast cancer management, managed based on FH	Consider RRSO at 45–50 years of age
*RAD51D*	Limited; potential increase in female breast cancer (including triple negative disease)	15–40	Ovarian cancer	Insufficient data for breast cancer management, managed based on FH	Consider RRSO at 45–50 years of age
*CDKN2A*	None	–	Pancreatic cancer Melanoma	–	Pancreatic cancer screening using contrast enhanced MRI/MRCP or EUS

*RRM *Risk reducing mastectomy, *RRBSO *Risk reducing bilateral salpingo-oophorectomy, *GIST *Gastro-intestinal stromal tumor, *TVUS *Transvaginal ultrasound,  *MRCP *Magnetic resonance cholangiopancreatography, *EUS *Endoscopic ultrasound, *FH *Family history

Data estimating the global incidence and prevalence of HBC comes predominantly from European populations and non-European High-Income Countries [10–20]. In Low Middle-Income Countries (LMICs), the population-specific risk of HBC in different ethnicities and the correlation with clinical characteristics remains largely unexplored.

### Previous studies in Pakistani population

There is little published data on HBC in the Pakistani population, and no study has reported the use of multi-gene Next Generation Sequencing (NGS) panel testing. A study using a two-tiered targeted sequencing approach on suspected high-risk patients with breast and/or ovarian cancer, found that 6.7% of patients tested positive, for *BRCA1*/*2* variants. Six out of 42 identified variants were found in multiple unrelated patients [21]. Rashid at el. found that 24.7% of high-risk breast cancer patients in their case series from Pakistan harbored a P/LP variant in *BRCA1*/2 [22]. In *BRCA1*, there were 18 recurrent variants and in *BRCA2* there were three recurrent variants found in unrelated families. In addition to these small insertions and deletions (INDELS) and single nucleotide variants (SNVs), 2.5% (*n* = 14/565) of high-risk patients had large genomic rearrangements (LGRs) [23, 24]. The same group further investigated the patients who did not harbor BRCA1/2 variants, sequentially for P/LP variants in *TP53, CHEK2, RAD51C*, and *PALB2*, using denaturing high-performance liquid chromatography (HPLC) analysis [25–28].

These studies reported pathogenic variants attributed to *TP53* in 0.95% (*n* = 1/105) and, *RAD51C* in 0.80% (*n* = 1/119) of the high-risk breast cancer patients. One nonsense pathogenic variant was identified in *PALB2*, accounting for 0.79% (*n* = 1/127) patients. Additionally, four in-silico predicted potentially deleterious variants, including three missense and one 5′ untranslated region variant were also identified in the study participants. Another study from the same group, that looked at the contribution of *CHEK2* in 374 breast and/or ovarian cancer patients and identified no known pathogenic variants. However, in 0.53% (*n* = 2/374) of participants, two missense in-silico predicted potentially deleterious variants were reported.

Another study, from Rashid at el. concluded that *RECQL*, which is a preliminary evidence breast cancer gene, was not a disease contributor in the studied series of cases [29].

However, as sequencing technology has advanced from single gene sequencing to high-throughput NGS platform based parallel gene sequencing, complemented by the progress in bioinformatics and variant classification, identification of P and LP variants in inherited breast cancer syndrome associated genes has become rapid and cost-effective. Thus, as multi-gene panel testing has now replaced sequential single gene testing, more patients are being identified to harbor variants in other breast-cancer risk genes. Studies report that about 4–16% test positive when they undergo multi-gene panel testing, after testing negative for *BRCA1/2* variants [25, 30–34].

In a previous paper, we described the challenges in establishing an HBC clinic at our academic medical center [35]. In this study, we report the results of using an expanded, multi-gene NGS panel for the first time in Pakistani patients and describe the spectrum of pathogenic variants found in a consecutive series of high-risk patients. We also explore unique genotype-phenotype correlations in the Pakistani population.

## Materials and methods

This is a retrospective review performed at a single Academic Medical Center in Karachi, Pakistan (Aga Khan University Hospital).

### Case selection

We selected patients who were seen in the HBC clinic and proceeded with multi-gene panel testing from May 2017 to April 2020. During this period of 36 months, multi-gene panel for breast cancer was sent for 284 individuals, referred from Breast Surgeons or Oncologists, from within AKUH and other hospitals in Karachi, including Liaquat National Hospital and Shaukat Khanum Memorial Cancer Hospital. Those who met the National Comprehensive Cancer Network (NCCN) referral criteria (NCCN 2016 version:1.2016), summarized in Table 2 were offered testing, and those who went ahead with testing were recruited for this study [36]. With regards to age at diagnosis, we divided our patients in age group brackets of 25–34, 35–44, 45–54, 55–64, and ≥ 65 years. Family history was considered positive if the patient had a close family relative (1st, 2nd or 3rd degree relative) with breast cancer or any other malignancies that may be part of a hereditary cancer syndrome, including ovarian, endometrial, small bowel, gastric, colorectal, pancreatic, prostate, brain, and thyroid cancer.

**Table 2:** NCCN guidelines for referral criteria (NCCN 2016 version 1.2016), used in the study [35]

Diagnosed with breast cancer at	< 50 years of age
Diagnosed with breast cancer at age 46–50 years with	Unknown or limited family history
	A second breast cancer at any age
	≥1 close blood relative with breast, ovarian, pancreatic, or prostate cancer at any age
Diagnosed with breast cancer at age ≤ 60 years with	Triple-negative breast cancer
Diagnosed at any age with	≥1 close blood relative with breast cancer at age ≤ 50 year
	Ovarian, pancreatic, metastatic, intraductal/cribriform histology, or high- or very-high risk group prostate cancer at any age
	≥ 3 total diagnoses of breast cancer in patient and/or close blood relatives
	Male breast cancer
	With epithelial ovarian cancer (including fallopian tube cancer or peritoneal cancer)
	Exocrine pancreatic cancer

Patients with ER or/and PR, score of 0–2 were considered as ER or/and PR negative, and those with ER or/and PR score ≥ 3 were considered as ER or/and PR positive. For HER2, IHC score of 0 or 1+ was considered as negative, a score of 2+ was considered as equivocal and a score of 3+ was considered as HER2 positive. For equivocal results, FISH (Fluorescence In Situ Hybridization) analysis was carried out, and if it was negative, then initial IHC score of + 2 for HER2 was counted as HER2 negative, otherwise FISH positive led to the initial IHC score of + 2 to be considered as HER2 positive.

### NGS assays

This testing was outsourced to one of two Clinical Laboratory Improvement Amendments (CLIA) and College of American Pathologists (CAP), certified commercial genetics laboratories based in the US; Invitae Genetics (*n* = 268) and Prevention Genetics (*n* = 16).

The NGS panel offered at Prevention Genetics included 27 genes, associated with hereditary breast cancer syndromes, while the panel offered at Invitae Genetics includes 37 genes, including 23 primary evidence and 14 preliminary evidence genes, associated with Hereditary Breast and Gynecologic cancers, summarized in Table 3 [37, 38].

**Table 3:** Summary of primary and preliminary evidence genes included in the multi-cancer panel at Invitae Genetics

Genes with guidelines (Primary evidence)	Genes without guidelines (Preliminary evidence)
*ATM, BRCA1, BRCA2, BRIP1, CDH1*	*ABRAXAS1, AKT1, CDC73, FANCC*
*CHECK2, EPCAM, MLH1, MSH2, MSH6*	*FANCM, MRE11, MUTYH, PIK3CA*
*NBN, NF1, PALB2, PMS2, PTEN*	*POLD1, RECQL, RINT1*
*RAD51C, RAD51D, STK11, TP53*	*SDHB, SDHD, XRC22*

The NGS platform makes uses of Illumina technology that offers full-gene sequencing and deletion/duplication analysis, with 99% analytical sensitivity and specificity for SNVs and insertions and deletions that are smaller than 15 base pairs as well as exon-level insertions and deletions. The sequence analysis covers the clinically important regions of the genes offered in the panels, including 10–20 base pairs in non-coding (intronic) regions of the selected gene transcript used.

The patient DNA is captured using a hybridization-based protocol, that is sequenced using Illumina’s Reversible Dye Terminator (RDT) platform and the reads are aligned to the reference sequence (GRCh37). The obtained results are confirmed using Sanger Sequencing at both laboratories. Additionally, Invitae Genetics utilizes other orthogonal technologies, including Pacific Biosciences SMRT sequencing, MLPA, MLPA-seq and Array CGH for NGS result validation, as needed. Thus, this testing is designed to detect SNVs, small INDELS, CNVs and LGRs [39].

### Variant classification

The variants identified (sequence finding) in the patients, after they are aligned to the reference sequence were then interpretations and assigned one of the five variant classifications as per ACMG guidelines: including Pathogenic (P), Likely Pathogenic (LP), Variants of Uncertain Significance (VUS), Likely Benign (LB) and Benign (B) [40].

### Statistical analysis

Data was obtained from patients who visited the HBC clinic, from May 2017 to April 2020. Descriptive and inferential statistics have been used to analyze clinical and histopathological characteristics of breast cancer patients with positive, VUS and negative genetic test results.

Fisher’s exact and Pearson’s chi-squared tests for categorical variables and Wilcoxon rank-sum test for quantitative variables were used. For comparison between two independent groups, Mann-Whitney test was performed. Results were considered significant at a *p* value of < 0.05. All statistical analyses were done using STATA 16.

### Study ethical approval

Ethical Review Approval Exemption (ERC) at the Aga Khan University was received for the retrospective chart review of the patients (insert ERC approval ID 2021–1332-18,429).

## Results

### Patient characteristics

Patient characteristics are shown in Table 4.

**Table 4:** Patient characteristics of the study participants

Study Characteristics Overview	*N* = number of patients	% of total patients
Total Study Participants	284	100.00
Males	5	1.76
Asymptomatic	1	0.35
Females	279	98.24
Asymptomatic	10	3.52
Unilateral	248	87.32
Bilateral	25	8.80
Asymptomatic	11	3.87
Breast and a secondary malignancy	5	1.76
Only breast	268	94.37
Asymptomatic	11	3.87
**Family History of Disease**		
Negative	75	26.41
Positive 1st Degree Relative for Breast Cancer Related Malignancy	106	37.32
Positive 2nd/3rd Degree Relative for Breast Cancer Related Malignancy	72	25.35
Positive 1-3rd Degree Relative for Non-Breast Cancer Related Malignancy	27	9.51
Incomplete Information	4	1.41

Out of 284 individuals, 273 patients were diagnosed with breast cancer and 11 asymptomatic individuals underwent testing, owing to their positive family history of breast cancer and/ovarian cancer. These 273 patients included four (1.46%) male breast cancer patients. The median age at diagnosis for all patients was 43 years (IQR: 36–50). Twenty-five of 273 (9.16%) patients had a diagnosis of bilateral breast cancer that included both synchronous and non-synchronous bilateral breast cancer. Five of 273 (1.83%) patients had a diagnosis of a second malignancy other than breast cancer, that included thyroid cancer (*n* = 2), ovarian cancer (*n* = 1), endometrial cancer (*n* = 1) and colon cancer (*n* = 1). No bilateral disease patients were recorded to have a second malignancy. Of the 11 asymptomatic individuals who underwent testing, one was male.

Histology was recorded for each tumor (*n* = 298), including unilateral (*n* = 248) and bilateral disease (*n* = 25). These included invasive ductal carcinoma (IDC) (*n* = 257, 86.24%), ductal carcinoma in situ (DCIS) (*n* = 22, 7.38%), invasive lobular carcinoma (ILC) (*n* = 9, 3.02%) and invasive papillary carcinoma (IPC) (*n* = 3, 1.10%). IHC was observed for each tumor recorded individually; from all unilateral and bilateral disease patients, (*n* = 298), out of which 71(23.83%) were triple negative breast cancer. (TNBC). Other IHC subtypes are summarized in Tables 5, 6, and 7.

**Table 5:** Immunohistochemistry subtypes summary

Study Characteristics	***N*** = number of tumors	% of total tumors
Breast Cancer (unilateral = 248, bilateral = 25)	298	100.00
**Grade**		
1	10	3.36
2	123	41.28
3	139	48.94
Unknown	26	8.72
**Histology**		
IDC	257	86.24
DCIS	22	7.38
ILC	9	3.02
Papillary	3	1.01
Unknown	7	2.35
**Immunohistochemistry**		
Triple Negative (−)	71	23.83
Triple Positive (+)	35	11.74
ER−/PR+/HER2+	2	0.67
ER−/PR+/HER2-	4	1.34
ER−/PR-	3	1.01
ER−/PR−/HER2+	24	8.05
ER+/PR−/HER2-	13	4.36
ER+/PR+/HER2-	115	38.59
ER+/PR−/HER2+	5	1.68
ER+/PR+	15	5.03
ER+/PR-	1	0.34
Unknown	10	3.36

**Table 6:** Pathogenic and likely pathogenic variants identified in *BRCA1* and *BRCA2*

Sr No.	Patient ID	Age at diagnosis	Type	Gene	Variant Details	Exon	Consequence	Reported in multiple unrelated families in our cohort (Family #)	Previously Reported in Pakistani Patients (no. of individuals)	Novel Variants	Published literature
	***BRCA1***										
1	256	35	U/L Breast Ca	*BRCA1*	c.68_69del (p.Glu23Valfs*17)	2	Frameshift	also reported in one unrelated ovarian cancer patient	Yes (1,1,1)	–	PMID: 12181777, 31,528,241, (Risch et al., 2001) [21, 22, 41]
2	22	36	U/L Breast Ca	*BRCA1*	c.1399_1453dup (p.Ala485Glufs*13)	10	Frameshift	–	Yes (1)	–	PMID:31528241 [22]
3	36	25	U/L Breast Ca	*BRCA1*	c.1450G > T(p.Gly484*)	10	Non-sense	–	–	–	–
4	51	34	U/L Breast Ca	*BRCA1*	c.685del (p.Ser229Leufs*5)	10	Frameshift	–	Yes (7)	–	PMID:31528241 [22]
5	179	21	U/L Breast Ca	*BRCA1*	c.895_896del (p.Val299Argfs*4)	10	Frameshift	179,191	Yes (1)	–	PMID:31528241 [22]
6	181	26	U/L Breast Ca	*BRCA1*	c.3607C > T (p.Arg1203*)	10	Non-sense	–	Yes (1)	–	PMID:31528241 [22]
7	191	36	U/L Breast Ca	*BRCA1*	c.895_896del (p.Val299Argfs*4),	10	Frameshift	179,191	Yes (1)	–	PMID:31528241 [22]
8	283	45	Asymptomatic proband	*BRCA1*	c.1583_1589del (p.Thr528Lysfs*2)	10	Frameshift				
9	194	37	U/L Breast Ca	*BRCA1*	c.3228_3229del (p.Gly1077Alafs*8)	10	Frameshift	–	–	–	
10	204	40	U/L Breast Ca	*BRCA1*	c.4065_4068del (p.Asn1355Lysfs*10)	10	Frameshift	–	Yes (4, 4, 1)	–	PMID: 12181777, 31,528,241, 8,571,953 [21, 22, 42]
11	220	31	U/L Breast Ca	*BRCA1*	c.3169_3172del, p.Ser1057Leufs*4,	10	Frameshift	–	Yes (2)	–	PMID:31528241 [22]
12	90	–	Asymptomatic proband	*BRCA1*	c.4485-1G > A	Intron 13	Splice acceptor	90, 145	Yes (6)	–	PMID:31528241 [22]
13	145	38, 47	B/L Breast Ca	*BRCA1*	c.4485-1G > A	Intron 13	Splice acceptor	90, 145	Yes (6)	–	PMID:31528241 [22]
14	223	32	U/L Breast Ca	*BRCA1*	c.4508C > A (p.Ser1503*)	14	Non-sense	223,282	Yes (3,1)	–	PMID: 12181777, 31,528,241 [21, 22]
15	282	40	U/L Breast Ca, Ovarian Ca	*BRCA1*	c.4508C > A (p.Ser1503*)	14	Non-sense	223,282	–	–	–
16	15	49	U/L Breast Ca	*BRCA1*	c.4821del, p.Ala1608Glnfs*25	15	Frameshift	–	–	Yes	–
17	82	30,43	B/L Breast Ca	*BRCA1*	c.5035del (p.Leu1679*)	16	Non-sense	82, 251	Yes (2)	–	–
18	251	30	B/L Breast Ca	*BRCA1*	c.5035del (p.Leu1679*),	16	Non-sense	82, 251	–	–	–
19	284	36	U/L Breast Ca	*BRCA1*	c.5074 + 1G > A	Intron 16	Splice donor	7284		–	PMID:31528241 [22]
20	7	27	U/L Breast Ca	*BRCA1*	c.5074 + 1G > A	Intron 16	Splice donor	7284	Yes, (2)	_	PMID:31528241 [22]
21	31	28	U/L Breast Ca	*BRCA1*	c.5176delA (p.Arg1726Glufs*4),	18	Frameshift	–	–	–	–
22	4	37	U/L Breast Ca	*BRCA1*	c.5278-1G > A	19	Splice acceptor	–	–	–	–
23	235	31	U/L Breast Ca	*BRCA1,*	c.5135G > A (p.Trp1712*),	17	Non-sense	–	–	–	–
				*CHEK2*	Gain (Exons 3–4)	Gain of Ex. 3–4	LGR				
24	13	31	U/L Breast Ca	*BRCA1*	Deletion (Exons 1–11)	Deletion of Ex.1–11	LGR	13,278	–	–	–
25	278	37	Asymptomatic proband	*BRCA1*	Deletion (Exons 1–11)	Deletion of Ex.1–11	LGR	13,278	–	–	–
26	28	32	U/L Breast Ca	*BRCA1*	Deletion (Exon 1–6)	Deletion of Ex.1–6	LGR	–		–	–
27	109	34	U/L Breast Ca	*BRCA1*	Deletion (Exon 23)	Deletion of Ex.23	LGR	–	–	–	–
28	63	33	U/L Breast Ca	*BRCA1*	Gain (Exon 3–11)	Gain of Ex.3–11	LGR	–	–	Yes	–
29	6	30	U/L Breast Ca	*BRCA1*	Deletion of Exons 1–2	Deletion of Ex.1–2	LGR	–	Yes, (7)	–	PMID:31528241 [22]
				*MSH6*	c.3261del (p.Phe1088Serfs*2)	5	Frameshift				
	***BRCA2***										
30	105	62	U/L Breast Ca	*BRCA2*	Deletion (Exon 3)	Deletion of Ex.3	LGR	**–**	–	–	**–**
				*ATM*	Deletion (Exon 43)	Deletion of Ex. 43	LGR				
31	45	53	U/L Breast Ca	*BRCA2*	c.426-2A > G	Intron 4	Splice acceptor	–	Yes (1)	–	PMID:31528241 [22]
32	257	35	U/L Breast Ca	*BRCA2*	c.859del (p.Met287Cysfs*5)	10	Frameshift	–	–	–	–
33	119	55	U/L Breast Ca	*BRCA2*	c.3109C > T (p.Gln1037*)	11	Non-sense	–	Found in Asian patient (4)		PMID: 19241424 [43]
34	1	52	U/L Breast Ca	*BRCA2*	c.4718del (p.Cys1573Leufs*6)	11	Frameshift	–	–	–	–
35	34	41	U/L Breast Ca	*BRCA2*	c.6444dupT (p.Ile2149Tyrfs*2)	11	Frameshift	–	–	–	–
36	185	36	U/L Breast Ca	*BRCA2*	c.4471_4474del (p.Leu1491Lysfs*12)	11	Frameshift	–	–	–	–
37	76	48	U/L Breast Ca	*BRCA2*	c.7806-1G > C	Intron 16	Splice acceptor	–	–	–	–
38	200	40, 55	B/L Breast Ca	*BRCA2*	c.9380G > A (p.Trp3127*)	25	Non-sense	–	–	–	–

**Table 7:** Pathogenic and likely pathogenic variants identified in genes other than *BRCA1* and *BRCA2*

Serial No.	Patient ID	Age at diagnosis	Type	Gene	Variant Details	Exon	Consequence
1	44	29	U/L Breast Ca	*ATM*	c.8480 T > G (p.Phe2827Cys),	58	Missense
2	135	58, 63	B/L Breast Ca	*BARD1*	Deletion (Entire coding sequence)	Exon 1–11	LGR
3	169	40	U/L Breast Ca	*BARD1*	Deletion (Entire coding sequence)	Exon 1–11	LGR
4	20	41	U/L Breast Ca	*CHEK2*	c.58C > T (p.Gln20*)	2	Non-sense
5	96	35	U/L Breast Ca	*CHEK2*	Deletion (Exon 5)	Deletion of Ex. 5	LGR
6	165	60	B/L Breast Ca	*CHEK2*	c.409C > T (p.Arg137*)	3	Non-sense
7	225	40	U/L Breast Ca	*CHEK2*	c.58C > T (p.Gln20*)	2	Non-sense
8	21	44	U/L Breast Ca	*CHEK2*	c.283C > T (p.Arg95*)	2	Non-sense
				*RAD51C*	c.701C > G (p.Ser234*)	4	Non-sense
9	243	56	U/L Breast Ca	*FANCM*	c.4153G > T (p.Glu1385*),	14	Non-sense
10	279	43	U/L Breast Ca	*FANCM*	c.2199_2202del (p.Ser734Asnfs*25),	13	Frameshift
11	11	43	U/L Breast Ca	*MLH1*	Deletion (Exon 16–19)	Deletion of Ex. 16–19	LGR
12	241	60	U/L Breast Ca	*MLH1*	c.1897-2A > G (Splice acceptor)	Intron 16	Splice acceptor
13	269	39	U/L Breast Ca	*MLH1*	c.306G > T (p.Glu102Asp)	3	Missense
14	102	30	U/L Breast Ca	*MSH6*	c.1222_1226del (p.Pro408Aspfs*8),	4	Frameshift
15	202	51	U/L Breast Ca	*MUTYH*	c.312C > A (p.Tyr104*)	3	Non-sense
16	248	48	U/L Breast Ca	*MUTYH*	c.312C > A (p.Tyr104*)	3	Non-sense
17	80	66	U/L Breast Ca	*NF1*	c.5205 + 5G > A	Intron 36	Splice donor
18	227	38	U/L Breast Ca	*PALB2*	c.2488del (p.Glu830Serfs*21),	5	Frameshift
19	258	42	U/L Breast Ca	*PALB2*	c.2353_2354del (p.Pro785Thrfs*16)	5	Frameshift
20	57	64	B/L Breast Ca	*PALB2*	c.2488del (p.Glu830Serfs*21)	5	Frameshift
21	18	34,37	B/L Breast Ca	*RAD51D*	c.898C > T, p.Arg300*,	9	Non-sense
22	144	55	U/L Breast Ca	*RAD51D*	c.620C > T (p.Ser207Leu)	7	Missense
				*FANCM*	c.4318-1G > A (Splice acceptor)	Intron 15	Splice acceptor
23	184	40	U/L Breast Ca	*TP53*	c.537 T > G (p.His179Gln)	5	Missense
24	240	29	U/L Breast Ca	*TP53*	c.437G > A (p.Trp146*)	5	Non-sense

This included all patients with unilateral (*n* = 62) and bilateral disease (*n* = 8). In the bilateral disease patient group, three patients had both tumors as triple negative (*n* = 6), and three had either of the two tumors as triple negative (*n* = 3), that along with 62 unilateral disease patients made a total of 71 TNBC tumors. In the DCIS and ILC and IPC, none of the tumors were found to be triple negative. The statistical analyses involving IHC subtypes were carried out on patients with unilateral disease with IDC histology.

### Multi-gene panel result distribution

**Fig. 1:**
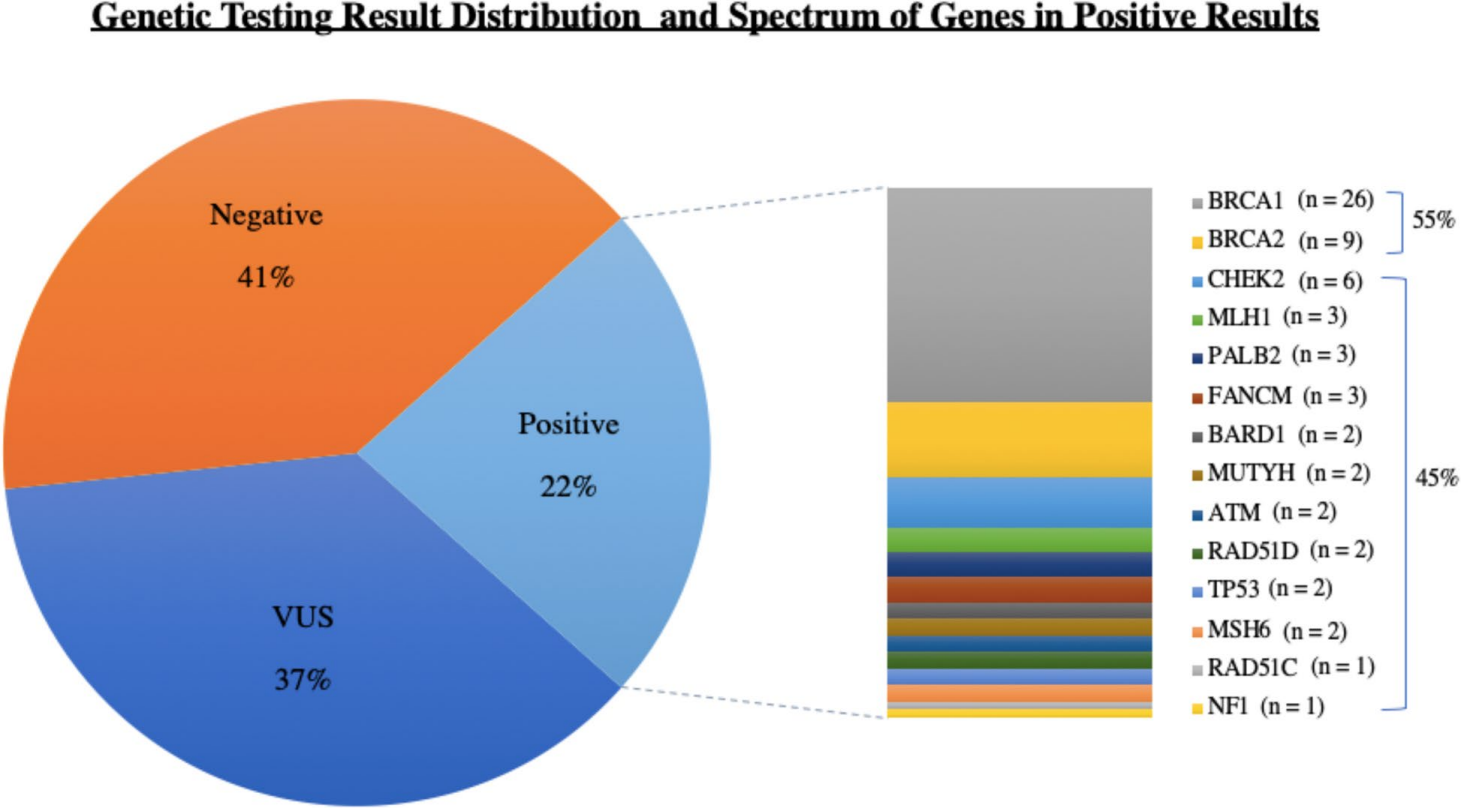
The spectrum of the identified variants is summarized, showing 45% of the positive results are attributed to non BRCA1/2 genes

Out of 273 patients, 59 (22%) harbored P/LP variant (positive result), 103 (37%) had a VUS and 111 (41%) did not harbor any P/LP variant or a VUS (negative result). Of the 59 patients that harbored P/LP variants, 5 had a P/LP variant in two genes. Out of these total 64 P/LP variants, 35 (55%) were in either *BRCA1* or *BRCA2*. The spectrum of the identified variants is summarized in Fig. 1.

Patients with P/LP variants on their genetic test had a younger age at diagnosis compared to those having a VUS and negative result; median age = 37.5, IQR (31.5–48) vs median age = 45, vs, median age = 44, (*p* = 0.002) respectively, using Mann-Whitney test. A larger percentage of results with P/LP variants were found in the youngest age group of 25–34 years (*n* = 19) and 35–44 years (*n* = 25), accounting for 74% (*n* = 44/59) of the positive genetic test results. Eleven of these 44 patients (25%) had a negative family history of breast or related cancers.

One hundred and sixty-seven of the 273 patients (61%) reported a positive family history. Forty-five of these 167 patients (27%) harbored a P/LP variant, compared to 15 out of 102 (15%) patients with a negative family history. Out of all patients who harbored a P/LP variant, (*n* = 15/60) 25% had a negative family history for breast or related malignancies.

We also analyzed if there was an association found between family history of disease and genetic test result outcomes. Having a positive family history was associated with having a positive test result (*p* = 0.019). However, as expected, the association of family history was not established in case of VUS (*p* = 0.780) and negative (*p* = 0.088) result outcomes. Furthermore, the likelihood of a genetic test being positive was two times higher in the presence of a family history of cancer as compared to no family history of cancer (OR = 2.13, CI = 1.12–4.08, *p* = 0.021).

In patients with unilateral disease who presented without a history of another malignancy; the diagnostic yield was 20% (*n* = 49/243). Out of them 20% (*n* = 11/50) presented with negative family history of disease. Five patients with unilateral disease also presented with a secondary malignancy. For these patients, the diagnostic yield was 40% (*n* = 2/5) and both patients had a positive family history of disease. For patients with bilateral breast cancer, the diagnostic yield was 32% (*n* = 8/25), out of whom 50% (*n* = 4/8) presented with a negative family history.

In patients with unilateral breast cancer who had a positive result, there was a significant association between having TNBC and having a positive genetic test result (*p* < 0.001). Additionally, logistic regression analysis showed that patients with TNBC were almost 3 times more likely to have a positive result (OR = 2.79, CI = 1.42–5.48 *p* = 0.003).

Sixty-two patients had TNBC, and 24 of these 62 patients had a positive result (38.7%). Twenty of these sixty-two TNBC patients (32.3%) were positive for *BRCA1/2* variants. Two of these 20 patients were also found to have pathogenic variants in a second cancer predisposition gene, *CHEK2* in one patient, and *MSH6* in the other (patient ID: 6 and 235 respectively). In the remaining four patients with TNBC who tested positive, two patients had di-genic pathogenic variants including, *CHEK2* and *RAD51C* in patient 21 and *RAD51D* and *FANCM* in patient 144. The other two patients had pathogenic variants in *PALB2* and *MLH1*. Overall, 38.7% of TNBC patients received a diagnosed of a hereditary breast cancer syndrome and out of those patients, (*n* = 18/24) 75% had a pathogenic variant in either *BRCA1* or *BRCA2* solely.

Alternatively, out of all patients who tested positive for *BRCA1/2 v*ariants, 21 of 35 (60%) had TNBC. *BRCA1/2* positive patients contributed towards only 6.41% of other non-TNBC tumor IHC subtypes (*n* = 10/156) using Fisher’s Exact test (*p* value = 0.004).

On the contrary, the association of tumor immunohistochemical subtypes of patients with VUS and negative results was statistically insignificant, (*p* = 0.710). Furthermore, no statistically significant association was found between having a positive test result and high-grade disease presentation (OR = 1.4, Cl = 0.79–2.47, *p* = 0.245). This association was also not statistically significant for *BRCA1* positive patients and high-grade disease (OR = 2.1, Cl = 0.70–6.40, *p* = 0.181) **(**Additional file 1**).**

## Discussion

To our knowledge, this is the first study to report the spectrum of germline pathogenic variants in genes beyond *BRCA1/2* identified in breast cancer patients from Pakistan, using an NGS based multi-cancer gene panel and correlating with clinical characteristics. We report a positivity rate of 22% in our patients, confirming a diagnosis for HBC. This is within but on the higher end of the range of positivity due to P/LP variants in *BRCA1/2 *variants in various other countries, where it is reported to be between 9.4 to 29.8% [11, 41–54]. However, most of these studies focused on *BRCA1/2* testing only and used different clinical criteria for testing. In our study, we used standardized NCCN criteria and expanded multi-gene NGS panel testing.

In previous studies from Pakistan in which only *BRCA1/2* variants were tested, positive results were obtained in 25% of high-risk families for breast and ovarian cancer syndrome. Rashid et al. identified nine variants that were specific to the Pakistani population, with 18 and three recurrent variants in *BRCA1* and *BRCA2* respectively [22, 23, 44, 45]. In our patients, we observed seven variants in *BRCA1* that had been previously reported by Rashid el al, that included c.68_69del, c.1399_1453dup, c.895_896del, c.4065_4068del, c.4508C > A, c.5074 + 1G > A and Deletion of Exons 1–2. Twenty-nine patients had pathogenic variants in *BRCA1*, harboring 23 unique variants including 6 variants that were found in multiple unrelated patients, which included c. 895_896del, c. 4485-1G > A, c.4508C > A, c.5035del, c.5074 + 1G > A and Deletion of Exon 1–11. One patient harbored c.3109C > T in *BRCA2*, which is a variant previously reported in Asian patients, but not specifically in Pakistani patients [46]. We also report two novel pathogenic variants in *BRCA1* that had not been previously reported in the literature, including Gain (Exon 3–11) and c.4821del.

Our work demonstrates that genes beyond *BRCA1/2* genes contributed to 45% (*n* = 29/64) of the positive results. This validates the utility of multi-gene panel testing over *BRCA1/2* only, improving the diagnostic yield by 8.8% (from *n* = 35/273 to *n* = 59/273) with multi-gene panel, comparable to what other studies have reported [29–33]. The diagnostic yield for unilateral disease was found to be 20%, while that for patients with a secondary malignancy was 40%, and for bilateral disease it was 32%, showing the predictive value of these two important clinical factors in identifying high-risk patients for testing. It also indicates that more work needs to be done to identify other genetic or non-hereditary biological factors in patients with bilateral disease, or breast cancer with an additional malignancy.

It is worth noting that out of 59 patients who had tested positive for genes present in the panel, five patients had disease-causing variants in multiple genes, (patient ID: 6, 21, 105, 144 and 235); in *BRCA1 and MSH6, CHEK2 and RAD51C, ATM* and *BRCA2, FANCM* and *RAD51D* and *BRCA1* and *CHEK2*; respectively. This further signifies the utility of multi-gene panel testing looking beyond *BRCA1/2* for optimal patient care. We will need to follow these patients over time to see if they follow a course that is more aggressive compared to patients with single pathogenic variants.

In our patient population, 26% (*n* = 15) patients who tested positive were over the age of 45. Out of the 44 patients who were below the age of 45 years at the time of diagnosis, 11 patients had a negative family history of breast or related cancers. This validates that age cut-off of 45 years as a stand-alone criterion for referral for germline testing as recommended by NCCN 2020 guidelines for our population as well. It is crucial to understand the importance of following recommended referral criteria. Otherwise, often the referring providers tend to use age cut-off of 45 plus a positive family history of disease to make the referrals to genetics clinic. If that was done in our clinic, then 26% patients would not have received their diagnosis of an HBC. In the larger group, we observed that positive family history doubles the likelihood of testing positive. Nonetheless, a quarter of patients testing positive had a negative family history of disease. This highlights the importance of offering germline testing to individuals even without a positive family history, as long as they meet other criteria for genetic testing. Making use of the phenotype and genotype information, we also attempted to evaluate the effect of updating our institutional testing criteria from NCCN guidelines 2016 to NCCN 2020 [9, 35]. We found that if the age cut-off as a stand-alone testing criterion would to be reduced from 50 years to 45 years, we would fail to establish HBC diagnosis in 1 (1.66%) of the study participants (*n* = 1). Additional file 2 discusses our patient stratification pipeline in detail.

We also observed that over a half of the patients with a VUS or a negative result had a positive family history. This may indicate the polygenic and multifactorial nature of breast cancer in those families, as well as the need to continue to identify novel monogenic causes of HBC that are currently not covered by commercially available multi-gene NGS panels [55, 56].

While reporting correlation between positive results and histopathological findings, we observed that the likelihood of a positive result across all genes was 3 times higher in TNBC (OR = 2.8, CI = 1.42–5.48 *p* = 0.003. The established association of *BRCA1/2* positive results with TNBC, was supported in our study population, with *BRCA1/2* positive patients contributed towards 32.3% cases with TNBC (*p* = < 0.001) [49, 50].

It was noted that other positive results in patients with TNBC included pathogenic variants in *CHEK2, FANCM, RAD51C, RAD51D, MLH1 and PALB2.* Four TNBC patients were identified to have di-genic pathogenic variants. Out of the genes that have a role in the *BRCA1/2* HR pathway **(**Additional file 3**)**, NCCN Guidelines (version 2.2021) indicate that the risk of triple negative breast cancer is potentially increased in patients with P/LP variants in *BRIP1*, *RAD51C* and *RAD51D* [9, 49, 50]. Couch et al. and Shimelis et al. also found association of TNBC with *BARD1* and *PALB2*, in addition to *BRIP1*, *RAD51C* and *RAD51D* [57, 58].

In patients with TNBC, a diagnosis of HBC was not established in 61.3% of patients. The diagnostic yield of 38.7% in our patients, however, was 2-folds higher than that reported by Couch et al. (14.6%) in a multicenter study cohort, unselected for family history of disease [40].

For patients who tested positive for *BRCA1*/2, 60% had TNBC; emphasizing that TNBC should not be seen as an isolated testing criterion; otherwise in about 40% cases, one would miss a diagnosis of HBC.

Studies have shown that *BRCA1* P/LP variant positive patients present with high-grade disease, compared to *BRCA2* P/LP variant positive patients and with sporadic/non-hereditary cases of breast cancer [59–65]. In our study, no statistically significant association was found in the analyzed subsets, and at large, the likelihood of getting a positive result with a higher disease grade was not established.

### Study limitations

Since the patients were referred to our centre from multiple institutions, we could not systemically capture the ethnicity details for the patients thereby, the ethnic diversity in the patient population cannot be objectively discussed. Given the fact that we are a referral center for the region, we believe that our patients do indeed represent the ethnic diversity present in the population. We will address this in future studies by documenting ethnicity information in our databases, as well as collaborating with multiple centers elsewhere in the country to ensure that we can objectively look at ethnic differences in the HBC population in our country.

**Fig. 2:**
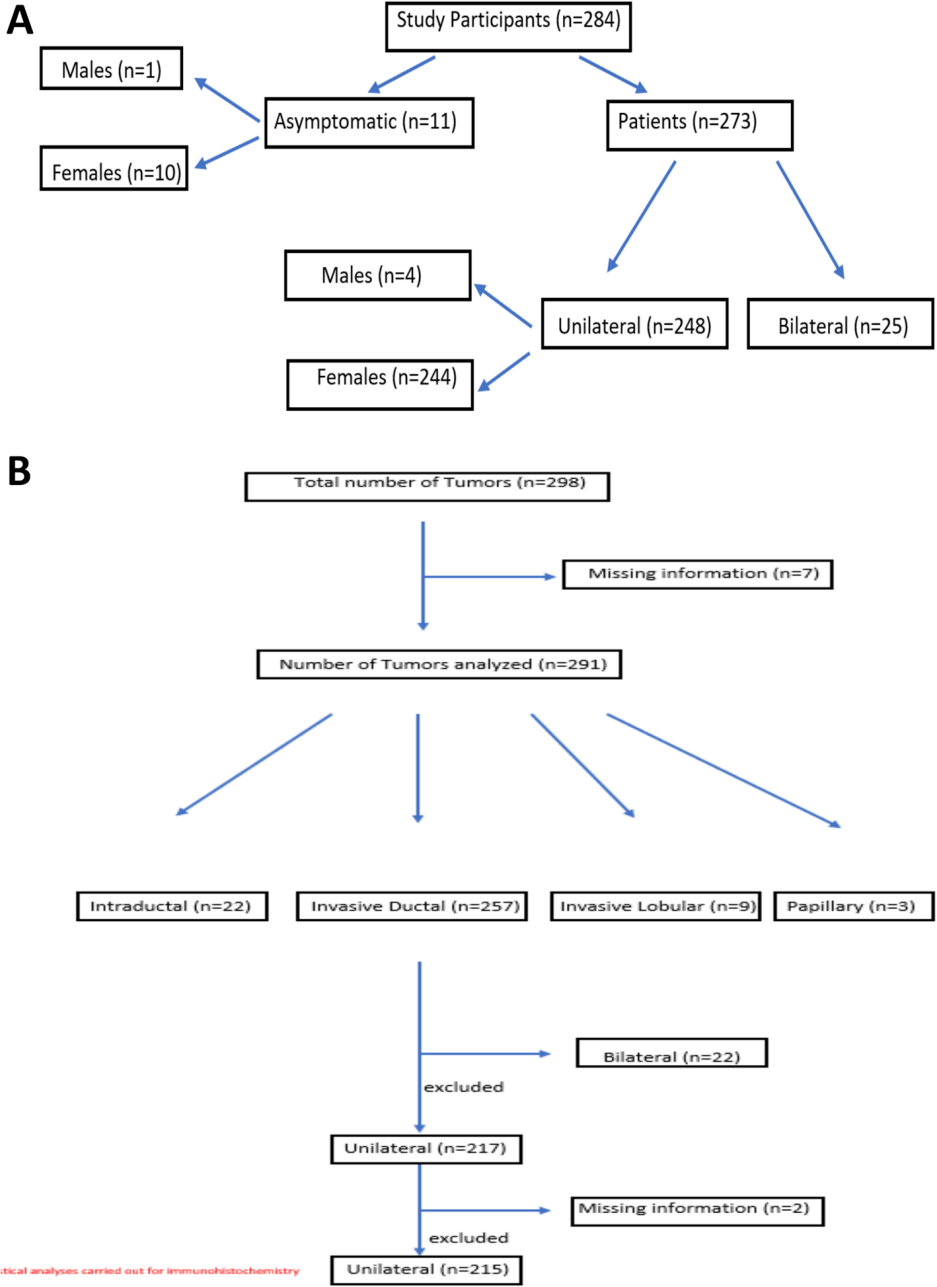
**A** Patient characteristics of the study participants (flow chart). **B** Patient Disease characteristics and triage framework of the study

In addition, of the 273 patients, the final histopathological statistical analysis was carried out on a total of 239 patients diagnosed with invasive ductal carcinoma (unilateral breast cancer *n* = 217 and bilateral breast cancer *n* = 22). Information on IHC subtypes for unilateral IDC cancer patients was available for (*n* = 215 out of 217), analysis carried out only on unilateral cancers, as bilateral cancer patients also included those with multiple possible IHC subtypes. The patient disease characteristics and triage framework is summarized in Figs. 2(A and B).

## Conclusion

In our HBC clinic, where NCCN guidelines were used to triage patients for genetic testing, the percentage of patients who tested positive was 22%, a rate on the higher end of what is reported in other populations.

The importance of expanded, multi-gene panel testing over just *BRCA1/2* testing is highlighted, as 41% (*n* = 24/59) of the patients had P or PL variants in genes other than *BRCA1/2*. Our test positivity rate (excluding the asymptomatic individuals) would have only been 12.8% (*n* = 35/273) if only *BRCA1/2* testing was done. We also observed that in patients with unilateral disease without a second malignancy, 20% were identified to have HBC. For unilateral patients with a secondary malignancy, and those with bilateral disease; 40 and 32% were identified to have HBC, respectively. Consistent with data from other countries, we also found that in our population, having TNBC or a young age at diagnosis increased the likelihood of a positive result. In over 60% of the patients with TNBC, the diagnosis of HBC was not established. Up to a quarter of patients with positive result had a negative family history. Over half of patients with a VUS or a negative result presented with a positive disease history.

To our knowledge, this is the first study from Pakistan to report the clinical utility and clinical correlation in patients who were tested through a multi-gene panel for HBC. As the database expands and protocol-driven referrals are made across the country, our insight about the genetic architecture of HBC in ethnically diverse Pakistani population will continue to increase. Subsequently, this will enable better elucidation of the underlying disease pathology to help devise improved preventive, diagnostic and therapeutic interventions to reduce breast cancer associated morbidity and mortality.

## Supplementary Information

**Additional file 1.** Presenting histopathological characteristics and Positive result association.

**Additional file 2.** Updating the institutional referral criteria from NCCN criteria 2016 to 2020.

**Additional file 3.** BRCA1/2 DDR and DNA repair pathway.

## Data Availability

The data and analyses performed to produce the result in this study can be made available by the corresponding author on reasonable request.

## Abbreviations

Array CGH: Array comparative genomic hybridization
B: Benign
CAP: College of American Pathologists
CLIA: Clinical Laboratory Improvement Amendments
CNV: Copy number variant
DCIS: Ductal carcinoma in situ
ER: Estrogen Receptor
EUS: Endoscopic ultrasound
FH: Family history
FISH: Fluorescence In-Situ Hybridization
GIST: Gastro-intestinal stromal tumor
HBC: Hereditary Breast Cancer
HER2: Human epidermal growth factor receptor 2
HPLC: High-performance liquid chromatography
IDC: Invasive ductal carcinoma
IHC: Immunohistochemistry
ILC: Invasive lobular carcinoma
INDELS: Insertions and deletions
IQR: Interquartile range
IPC: Invasive papillary carcinoma
LB: Likely Benign
LGRs: Large genomic rearrangements
LMIC: Low middle-income country
LP: Likely pathogenic
MLPA: Multiplex ligation-dependent probe amplification
MLPA-seq: Multiplex ligation-dependent probe amplification sequencing
MRCP: Magnetic resonance cholangiopancreatography
NCCN: National Comprehensive Cancer Network
NGS: Next Generation Sequencing
P: Pathogenic
PR: Progesterone receptor
RDT: Reversible dye terminator
RRM: Risk reducing mastectomy
RRBSO: Risk reducing bilateral salpingo-oophorectomy
SNV: Single nucleotide variant
TNBC: Triple negative breast cancer
TVUS: Transvaginal ultrasound
VUS: Variant of uncertain significance
