# Supplementary material for: Spectrum of germline pathogenic variants using a targeted next generation sequencing panel and genotype-phenotype correlations in patients with suspected hereditary breast cancer at an academic medical centre in Pakistan
Source: Hered Cancer Clin Pract. 2022 Jun 16;20:24. doi: 10.1186/s13053-022-00232-2 (PMC9204946; doi:10.1186/s13053-022-00232-2)
Supplement: Supplementary file 1 — Additional file 1. Presenting histopathological characteristics and Positive result association. [file 13053_2022_232_MOESM1_ESM.docx]

**Additional file 1**

*Presenting histopathological characteristics and Positive result association*

To determine possible association between positive results and presenting histopathological characteristics of the tumor analysis was carried out for all patients with a positive test result, and separately for *BRCA1* and *BRCA2* positive patients, compared to those who tested positive for other genes. Furthermore, all the positive results were compared against VUS and negative results, to understand if proven HBC presented with a higher-grade disease when compared with apparently sporadic/non-hereditary breast cancer. No statistically significant association was found, when the association of positive across all genes were analyzed against high grade disease presentation (OR=1.4, Cl=0.79 - 2.47, p = 0.245). The association was not statistically significant for *BRCA1* positive result patients presenting with high grade disease (OR=2.1, Cl=0.70-6.40, p = 0.181).
