# Supplementary material for: Spectrum of germline pathogenic variants using a targeted next generation sequencing panel and genotype-phenotype correlations in patients with suspected hereditary breast cancer at an academic medical centre in Pakistan
Source: Hered Cancer Clin Pract. 2022 Jun 16;20:24. doi: 10.1186/s13053-022-00232-2 (PMC9204946; doi:10.1186/s13053-022-00232-2)
Supplement: Supplementary file 2 — Additional file 2. Updating the institutional referral criteria from NCCN criteria 2016 to 2020. [file 13053_2022_232_MOESM2_ESM.docx]

**Additional file2**

*Updating the institutional referral criteria from NCCN criteria 2016 to 2020*

The NCCN criteria had changed over the years and in the latest NCCN criteria (2020), the criteria for testing based on age of diagnosis as a stand-alone testing qualifier had been reduced from 50 years to 45 years. During this work, we had followed the NCCN criteria (2016). Based on all the clinical and family history details, it was analyzed if adapting to the latest criteria will ensure if we are not missing any patient, testing positive for a germline disease-causing variant.

Out of 273 patients tested, 125 was at the age of over 45 years. Out of them, 20 tested positive (16.0% n=20/125 of total patients over the age of 45 years, 33.33% n=20/60 of who tested positive). Out of these 20 patients, 13 had a unilateral disease, without a secondary disease and 7 had a bilateral disease, the latter group would qualify for testing, based on additional testing criteria for bilateral disease. Out of the 13 patients, three had a TNBC and 10 had other immunohistochemistry subtypes; the former group would qualify for testing, based on additional testing criteria for TNBC testing upto 60 years. Out of the remaining 10 patients, nine patients had a positive family history of disease, and one patient had a negative family history of disease, the former group would qualify for testing based on additional testing criteria for positive family history of disease. Subsequently, based on this, it is observed that, one out of 60 patients (1.66%, testing positive, would be missed if the age cut-on has a stand-alone testing criterion would be reduced from 50 years to 45 years).

Patients over 45 at time of diagnosis: 125/273

Patients over 45 with a positive result: 20/125

Unilateral: 13 Bilateral: 7

(Qualify for testing based on bilateral disease)

TNBC: 3 Others: 10

(Qualify for testing based on TNBC disease age cut-off 60)

Positive FH: 9 Negative FH: 1 (1.66 %)

(qualify for testing based on FH) **would be missed out if age cut-off is 45**
