# Supplementary material for: Spectrum of germline pathogenic variants using a targeted next generation sequencing panel and genotype-phenotype correlations in patients with suspected hereditary breast cancer at an academic medical centre in Pakistan
Source: Hered Cancer Clin Pract. 2022 Jun 16;20:24. doi: 10.1186/s13053-022-00232-2 (PMC9204946; doi:10.1186/s13053-022-00232-2)
Supplement: Supplementary file 3 — Additional file 3. BRCA1/2 DDR and DNA repair pathway. [file 13053_2022_232_MOESM3_ESM.docx]

**Additional file 3**

*BRCA1/2 DDR and DNA repair pathway*

*BRCA1* and *BRCA2* are involved in DNA damage response (DDR) and DNA repair during the S and G2 phase in cell cycle, this is done by mediating homologous repair (HR) to maintain replication fidelity and have a role in tumorigenesis. *BRCA1* functions as DDR protein at two levels, involved in checkpoint activation and DNA repair. On the contrary, the role of *BRCA2* is at a later stage, and it functions as a mediator of core homologous recombination (HR) mechanism. HR is a crucial DNA repair process that makes use of the functional/undamaged sister chromatid to repair replication-associated DNA double-strand breaks (DSBs) through high-fidelity repair, that is a central genome integrity protection mechanism in proliferating cells. Loss of the protein function of *BRCA1* and *BRCA2* or other DDR mediators have a role in tumorigenesis. Pathogenic variants in *ATM* or *CHEK2*; that are DDR kinases also increases the hereditary cancer predisposition. Moreover, *CHK2* dependent phosphorylation of S988 in required for *BRCA1-PALB2-BRCA2* effector complex, that is needed for *RAD51* mediated HR. Other *BRCA1/2* HR pathway genes including the partner and localizer of *BRCA2, PALB2* and *BRIP1* as a *BRCA1*-interacting protein C-terminal helicase 1 are also associated with HBC, involved in *BRCA1/2* HR pathway. The *BRCA1-PALB2-BRCA2* complex is subsequently important to mediate *RAD51*-dependent HR. (47)(48)
